# Relationship between semen parameters, serum InhB, and INSL-3 levels, and the degree of varicocele

**DOI:** 10.1016/j.clinsp.2024.100339

**Published:** 2024-02-07

**Authors:** Lei Liu, WenJie Huang, KeBing Luo, YiZhou Zeng, YunHao Shao, ZongMin Long

**Affiliations:** Department of Urinary Surgery, The First people's hospital of Zunyi City (The third affiliated hospital of Zunyi Medical University), Zunyi, Guizhou Province, China

**Keywords:** Varicocele, Serum INSL-3, Semen parameters, Disease severity, Hormone

## Abstract

**Background:**

Varicocele is an abnormal expansion of the pampininias venous plexus in the scrotum, resulting in impaired sperm production and reduced sperm quality. The exact pathophysiological mechanism leading to varicocele-related infertility has not been fully elucidated. Although treatable, varicocele may lead to male infertility.

**Objective:**

To investigate the relationship between semen parameters, serum InhB and INSL-3 levels, and the degree of varicocele in male patients.

**Methods:**

Serum InhB and INSL-3 were detected. To evaluate the relationship between semen parameters and serum InhB and INSL-3 levels. To evaluate the value of semen parameters and serum InhB and INSL-3 levels in distinguishing disease severity in patients with varicocele.

**Results:**

Serum INSL-3 in patients with varicocele decreased with the severity of the disease. Serum INSL-3 was positively correlated with total sperm count and frequency of normal sperm morphology. There was a weak correlation between serum InhB and semen volume, concentration, and total sperm. Patients with different disease severity were similar within the groups, with partial overlap or similarity between varicocele Grade I and Grade II, and significant differences between Grade III and Grade I and II. Semen volume, concentration, total sperm, normal sperm morphology, and serum InhB and INSL-3 levels could distinguish the degree of varicocele.

**Conclusion:**

Semen parameters and the combination of serum InhB and INSL-3 levels in patients with varicocele are closely related to the severity of the disease. Serum INSL-3 is expected to be a potential biomarker for early clinical intervention.

## Highlights

• Serum INSL-3 in patients with varicocele decreases with the severity of the disease.
• Serum INSL-3 is positively correlated with total sperm count.
• Serum InhB is associated with semen volume, concentration, and total sperm.
• Serum InhB and INSL-3 levels could distinguish the degree of varicocele.

## Introduction

Varicocele is an abnormal expansion of the pampininias venous plexus in the scrotum, resulting in impaired sperm production and reduced sperm quality.^1^ The exact pathophysiological mechanism leading to varicocele-related infertility has not been fully elucidated. Although treatable, varicocele may lead to male infertility.^2^ Treatment options for varicocele include surgery or radiation therapy, but the safest and most effective treatment is still unclear. Therefore, a non-invasive marker is urgently needed to accurately analyze the spermatogenic function and sperm state in patients with varicocele.^3^

In recent years, statin B (InhB) has received increasing attention in the field of male clinical treatment.^4^^,^^5^ InhB is a heterodimeric glycoprotein mainly produced by Sertolis cells, which can inhibit the production and secretion of Follicle-Stimulating Hormone (FSH) and is closely related to spermatogenesis.^6^ InhB has endocrine, paracrine, and autocrine regulatory effects on reproductive function^7^ and is a marker of seminiferous tubule function. Manzoor et al. showed that serum InhB was positively correlated with sperm count and reflected the relationship between the function of Sertoli cell function and spermatogenesis.^8^ Grunewald et al. found that total motile sperm count was negatively correlated with FSH level, while InhB level was positively correlated with testicular volume.^5^ These findings may explain the clinical manifestations of impaired semen parameters in men with varicocele and reduced InhB levels.

Insulin-like 3-peptide (INSL-3) is a member of the insulin-like peptide superfamily, which is mainly produced in testicular stromal cells and theca cells, but the circulating hormone level in males is much higher than that in females.^9^ The INSL-3/RXFP2 system was initially discovered to exert in descensus testis during fetal development^10^ and has since been increasingly used to assess the function of intra-testicular interstitial cells, dependent on or independent of factors affecting the hypothalamus-hypophysis-gonadal axis.^11^ INSL-3 represents a different endpoint on the gonadal axis that interacts with a specific receptor of RXFP2, which acts on the interstitial cells themselves to regulate the production of steroid hormones, as well as on male germ cells.^12^

To date, most clinical studies have independently investigated semen parameters, blood biomarkers, and hormones in patients with varicocele. To our knowledge, no clinical studies have investigated serum INSL-3 levels in different patients with varicocele. Based on this, the authors boldly assumed that InhB and INSL-3 were related to spermatogenesis and semen quality, and semen parameters were related to the ability of serum InhB and INSL-3 levels to distinguish varicocele.

## Materials and methods

### Patients

The study was carried out and reported in line with the recommendations set out in the STARD 2015 guidance for diagnostic test reporting. The prospective study was conducted from January 2021 to March 2023. Patients who were admitted to The Third Affiliated Hospital of Zunyi Medical University for scrotal discomfort. Detailed inquisition, physical examination, scrotal Doppler ultrasonography, and measurement of serum FSH and total testosterone levels were performed in patients with suspected varicocele. The physical examination was conducted and graded by two physicians trained in the physical management of varicocele.

Inclusion criteria

(1) Men between the ages of 18 and 35 years at the time of medical examination; (2) Men clinically diagnosed as varicocele by physical examination and confirmed by Doppler ultrasound. Grade I: no abnormality in scrotal palpation in a calm state, and varicose spermatic vein could be touched by the VALSALVA test. Grade II: varicose spermatic vein can be touched in a calm state, but not visible on the scrotal surface; Grade III: Varicocele mass can be seen on the scrotal surface and felt by palpation. (3) Non-smokers. (4) No history of varicocele surgery, genitourinary infection, or antioxidant treatment in the past 6-months.

Exclusion criteria

Patients with azoospermia and leukocytospermia in semen analysis.

A total of 121 men met the inclusion criteria and were set as a patient group. Furthermore, the study incorporated a cohort of 20 control populations exhibiting semen parameters within the normal range as defined by the standards set forth by the World Health Organization.^13^

### Ethical statement

This study protocol was reviewed and approved by the Institutional Review Committee of The Third Affiliated Hospital of Zunyi Medical University (n° 20200612ZY). All subjects submitted informed consent upon enrollment.

### Semen analysis

After four to seven days of abstinence from sex, semen samples obtained through masturbation were collected in sterile containers. After liquefaction, semen samples were evaluated for semen volume, morphology, and viscosity. Semen characteristics were examined according to the 2010 World Health Organization standards.

### Hormone detection

Beckman DXI800 automatic chemiluminescence analyzer, reagents, standards, and calibration liquid were provided by Beckman. The quality control serum was adopted from BIO-RAD Company. The venous blood of all the tested subjects was extracted on an empty stomach in the morning, and the serum was separated within 30 min. Intra-assay and inter-assay variations were less than 5 %. Normal reference value range: FSH: 1.4‒15.2 mIU/mL; Testosterone: 2.6‒30.2 nmol/L.

### Serum InhB and INSL-3

All the tested subjects had venous blood drawn on an empty stomach in the morning. The sample was centrifuged, and serum was absorbed and frozen in a -80 °C refrigerator. Serum InhB and INSL-3 levels were detected by double-antibody ELISA kits (KHB Company, Shanghai, China).

### Statistical analysis

The variable distribution was evaluated using the Kolmogorov-Smirnov test, with the result expressed as a median [quartile]. Mann–Whitney *U* or Kruskal–Wallis test compared two or more sets of independent samples. Spearman's method calculated the correlation between variables, and *p* < 0.05 was analyzed by Benjamini–Hochberg False Discovery Rate (FDR). Adjusted *p* < 0.05 was statistically significant. G*power program (University of Dusseldorf, Germany) calculated the sample size of the study, two-tailed *t*-test had an effect size of 0.8 and α-error of 0.05. Two-tail tests assessed statistical significance, and *p* < 0.05 was considered to indicate statistical significance. Statistical tests were performed using IBM SPSS version 22.0 and graphs were produced using GraphPad Prism 9.0.

## Results

General characteristics and statistical data of the study population are shown in Table 1. Of the 121 patients, 4 (3.3 %) had comorbidities, 3 had a history of high blood pressure, and 1 had a history of diabetes. In the cohort of patients with varicocele, 16 (13.22 %), 43 (35.54 %), and 62 (51.24 %) patients were clinically diagnosed as Grade I, II, and III, respectively. There were 85 unilateral patients, up to 70.25 % of the study cohort.

**Table 1:** Serum InhB and INSL-3 in control group and varicoceles group.

Group	Control (*n* = 20)	Grade Ⅰ (*n* = 16)	Grade Ⅱ (*n* = 43)	Grade Ⅲ (*n* = 62)	*p*-value
Serum InhB (pg/mL)	184.6 [179.5‒192.7]	156.7 [148.8‒192.7]^a^	151.7 [143.0‒158.1]^a^	140.9 [134.0‒145.4]^a^	<0.001
Serum INSL-3 (pg/mL)	369.5 [390.8‒413.5]	286. [277.3‒290.7]^a^	272.4 [263.7‒279.6]^a^	247.3 [241.0‒255.8]^a^	<0.001

The Kruskal Wallis test was used to compare four independent samples, *p*-value < 0.05 was considered statistically significant. Using Mann Whitney U test to test the differences between two independent samples, the control group and Grade I/II/III group.

a *p*-value < 0.001.

### Semen parameters and hormone analysis

Semen parameters, except pH, semen volume, concentration, total sperm count, normal morphology (%), and total motility (PR+NP) were lower in men with varicocele than in healthy control subjects (Table 2). In addition, patients with varicocele had lower testosterone and FSH than healthy subjects (*p* < 0.001; *p* = 0.048) (Table 2).

**Table 2:** General characteristics and statistical data of the study population.

Variable	Control (*n* = 20)	Varicocele (*n* = 121)	*p*-value
Age (y)	24 [22‒30]	25 [22‒32]	
Hypertension		3 (2.48)	
Diabetes		1 (0.83)	
Grade			
Ⅰ		16 (13.22)	
Ⅱ		43 (35.54)	
Ⅲ		62 (51.24)	
Laterality			
Unilateral		85 (70.25)	
Bilateral		27 (22.31)	
Sperm parameters			
pH	7.2 [7.2‒7.5]	7.2 [7.0‒7.5]	0.805
Semen volume (mL)	3.0 [2.6‒4.3]	2.9 [2.7‒3.6]	<0.001
Concentration (106 sperm/mL)	52.3 [36.5‒69.6]	39.6 [35.2‒44.2]	<0.001
Total sperm number (106)	158.6 [135.6‒235.5]	120.8 [90.7‒156.1]	<0.001
Morphology (%normal)	5.8 [4.6‒8.6]	1.84 [1.22‒2.38]	<0.001
Motility (PR + NP) %	59 [55‒72]	45 [41‒50]	<0.001
Testosterone (nmoL/L)	15.32 [12.68‒27.65]	7.4 [6.8‒8.3]	<0.001
FSH (mIU/mL)	6.2 [5.5‒11.4]	8.4 [5.9‒11.4]	0.048
Serum InhB (pg/mL)	183.8 [179.6‒192.2]	145.6 [135.5‒157.0]	<0.001
Serum INSL-3 (pg/mL)	396.2 [390.8‒412.1]	260.0 [246.8‒274.9]	<0.001

### Relationship between the degree of varicocele and serum InhB and INSL-3

**Fig. 1:**
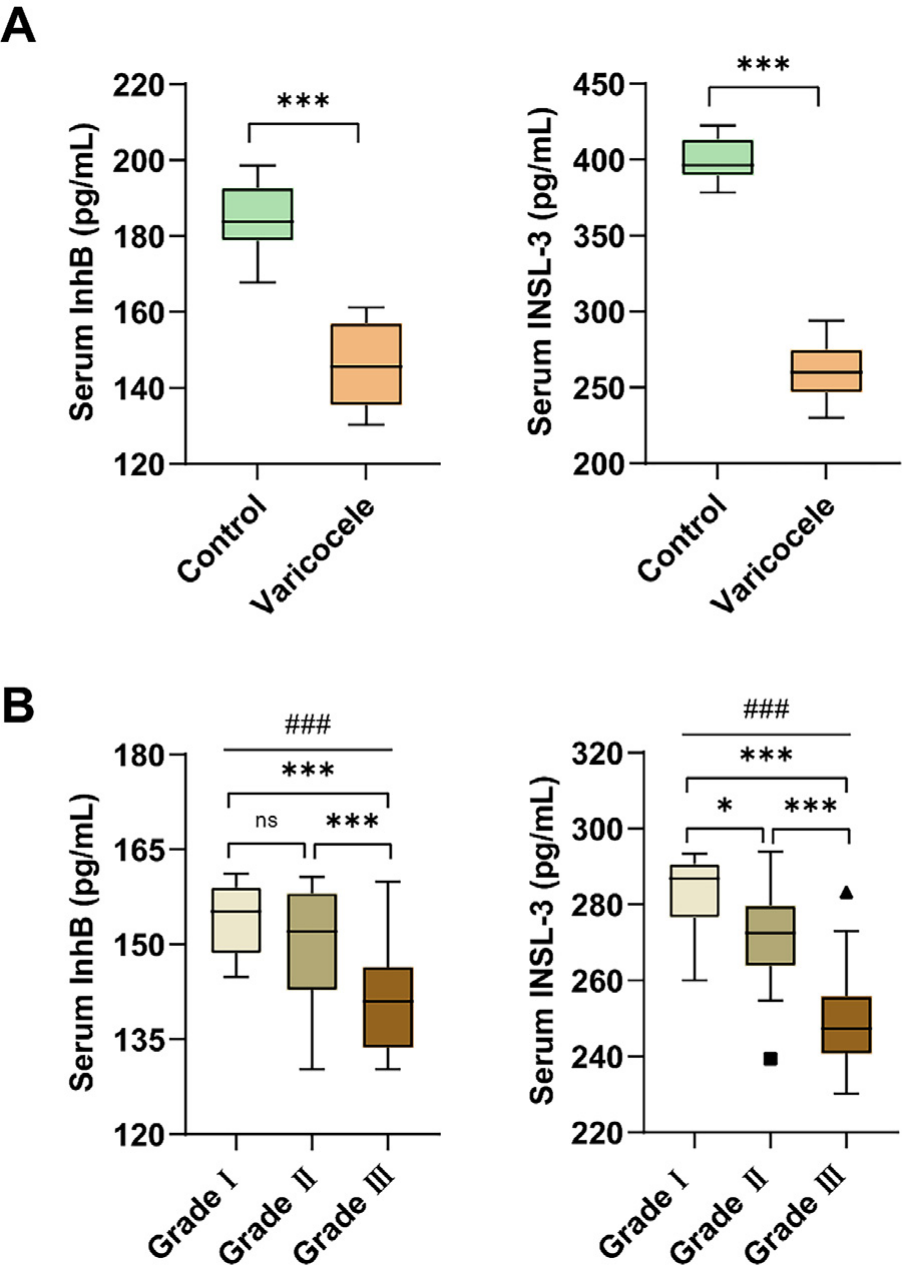
*Serum InhB and INSL-3.* Serum InhB and INSL-3 in control group and varicoceles group (A) and patients with varying degrees of varicocele (B). Data were expressed as the median (IQR) and compared by Mann–Whitney *U* or Kruskal–Wallis H test. Comparison between two groups, *** *p* < 0.001; ** *p* < 0.01; * *p* < 0.05; Multiple group comparisons, ### *p* < 0.001.

Notably, patients had lower serum InhB and INSL-3 compared to healthy subjects (*p* < 0.001) (Table 2, Fig. 1A). Further, serum InhB and INSL-3 were correlated with the degree of varicoceles, and serum InhB and INSL-3 decreased with the severity of the disease, especially serum INSL-3 (Fig. 1B).

### Semen parameters, serum InhB and INSL-3 levels, and the degree of varicocele

**Fig. 2:**
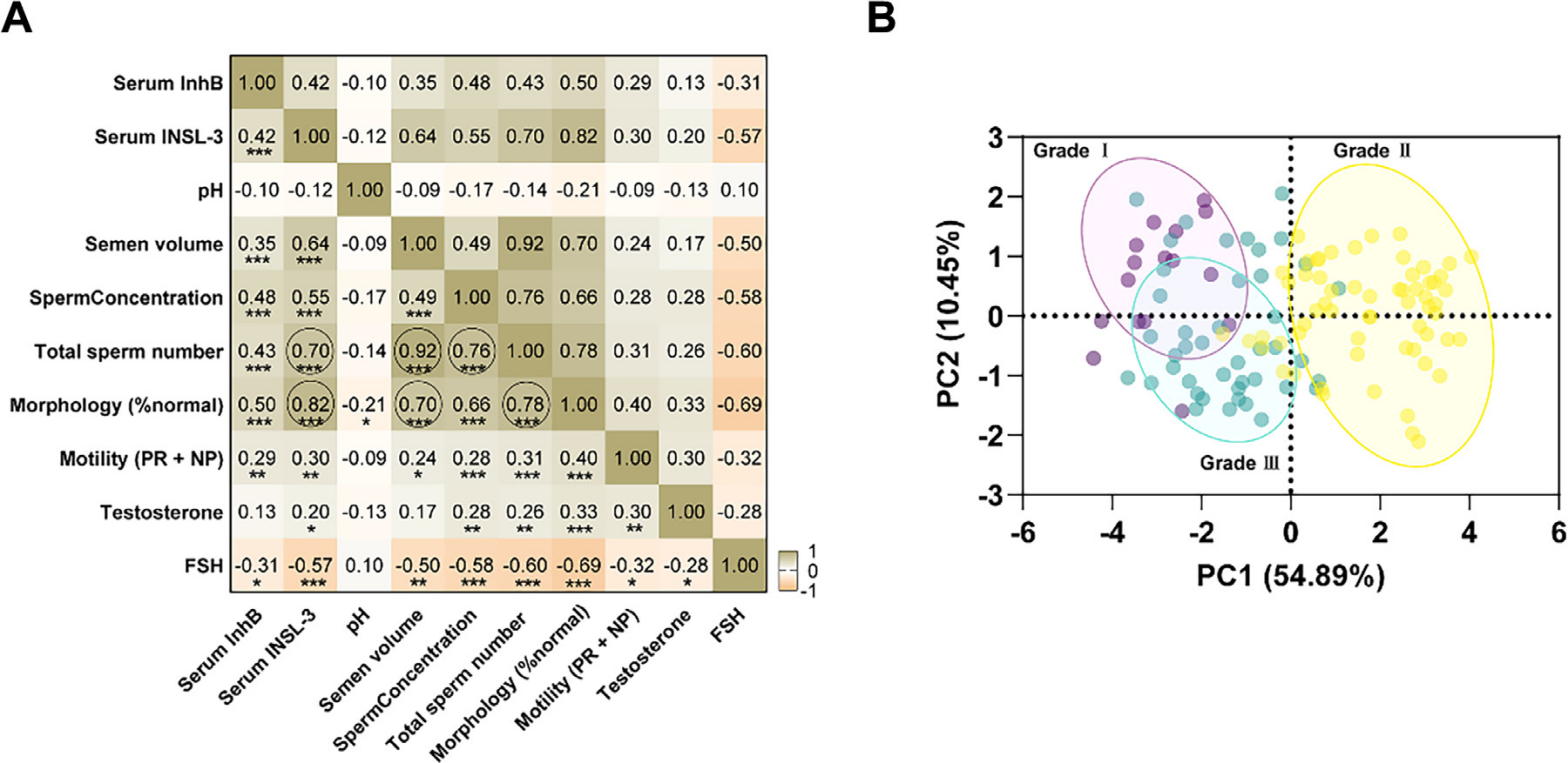
*Correlation analysis of serum InhB, INSL-3, and semen parameters.* Spearman's correlation coefficient analyzed the association among factors, The correlations were assessed by means of Spearmans coefficient (*r*): *r* = 0.3‒0.5 poor correlation, *r s* = 0.5‒0.7 middling correlation, *r* > 0.7 strong correlation, and all paired-comparison p values (*p* < 0.05) were compared by Benjamini-Hochberg FDR analysis to calculate the corrected p-values (A); unsupervised Principal Component Analysis (PCA) chart, Abbreviations: PC1, Principal Component 1; PC2, Principal Component 2 (B). *** *p* < 0.001; ** *p* < 0.01; * *p* < 0.05.

Spearman correlation analysis was performed to test the relationship between semen parameters and serum InhB and INSL-3 levels. As shown in Fig. 2A, FSH had a weak negative correlation with serum InhB, INSL-3, testosterone, and semen parameters. More positive correlations were shown between different variables. As expected, total sperm count showed a strong positive correlation with semen volume and sperm concentration (*r* = 0.92, *p* < 0.001; *r* = 0.76, *p* < 0.001) (Fig. 2A). In addition, the frequency of normal sperm morphology was significantly positively correlated with semen volume and total sperm count (*r* = 0.70, *p* < 0.001; *r* = 0.78, *p* < 0.001) (Fig. 2A). It was noted that serum INSL-3 was positively correlated with total sperm count and frequency of normal sperm morphology (*r* = 0.70, *p* < 0.001; *r* = 0.82, *p* < 0.001) (Fig. 2A). Serum InhB showed a weak positive correlation with most semen parameters, including semen volume, semen concentration, total sperm count, and serum INSL-3, while FSH has a weak negative correlation with serum InhB and INSL-3 levels and semen parameters (*r* = 0.31, *p* < 0.05; *r* = 0.57, *p* < 0.001) (Fig. 2A).

**Fig. 3:**
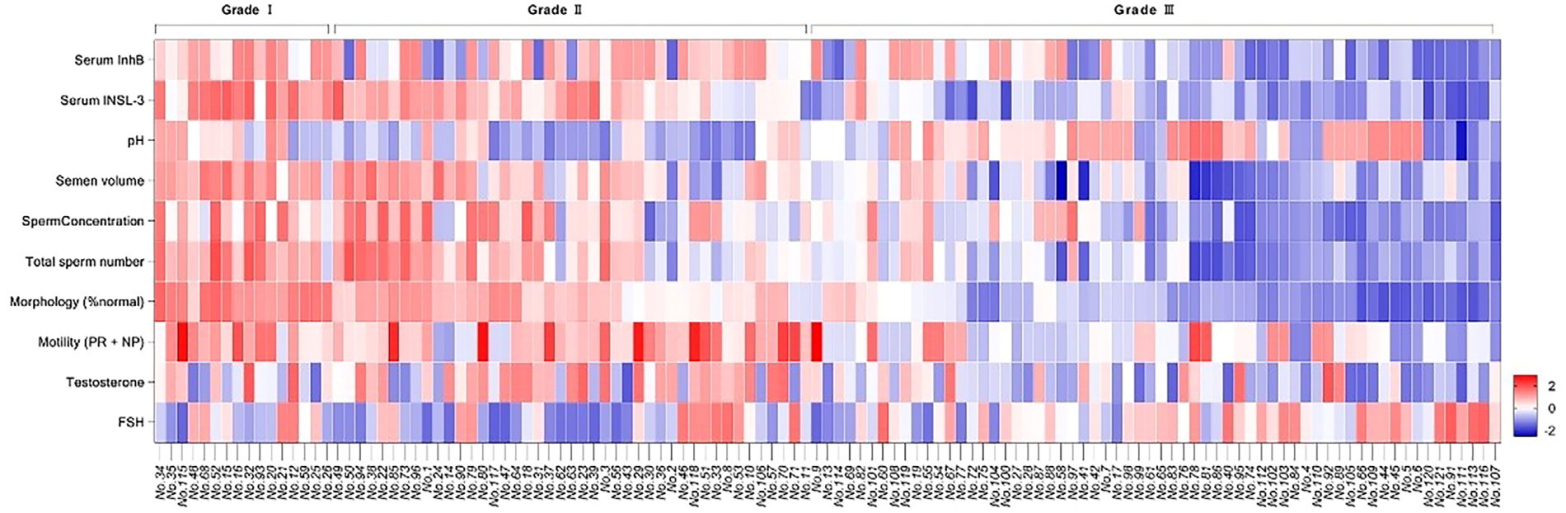
*Analysis of serum InhB, INSL-3, and semen parameters on the degree of varicocele.* Data were treated by Z-Score standardized heat maps. Z-score = [(control mean) - (individual value)] / (control SD). The Z-score serves as an indicator of the extent of association between the patient and the parameter, with a higher absolute value indicating a stronger correlation. Conversely, a lower absolute value signifies a weaker correlation. A Z-score of 0 denotes the absence of correlation.

Next, Unsupervised clustering was performed by principal component analysis (PCA) of varicocele severity. Combined with serum InhB, INSL-3 levels, and semen parameters, showed that patients of the same severity tended to cluster within the group (Fig. 2B). Grade III varicocele was well-separated from Grade I and Grade II. However, there was more overlap between Grade I and Grade II. The eigenvalue of principal component 1 was greater than 1 and the variance contribution rate of the two principal components was 54.89 %. The higher contribution rate of principal component 1 was mainly due to variables with an absolute load coefficient greater than 0.6, including serum INSL-3, semen volume, semen concentration, total sperm count, and normal sperm morphology (%). Heatmaps show scaled (z-score) expression values and showed that Grade III varicocele could be stratified well with Grade I and Grade II (Fig. 3). Although patients with varicocele had significantly lower testosterone than healthy controls, no significant stratification was seen among Grades III, II, and I (Fig. 3).

## Discussion

The authors found that the serum InhB and INSL-3 in patients with varicocele were higher, and the levels decreased with the severity of the disease. To our knowledge, this is the first study to demonstrate that the degree of disease in patients with varicocele is correlated with serum InhB and INSL-3 levels. In fact, the literature on serum InhB in male clinical use is a valuable potential biomarker to distinguish infertile patients from fertile patients.^14^^,^^15^ INSL-3 is more clinically associated with descensus testis in males.^16^ AlAli Badereddin et al. studied 716 young patients with varicoceles in Australia and found asthenospermia (17.9 %), oligo asthenospermia (14.2 %), and oligospermia (13.2 %), suggesting a direct relationship between the degree of varicoceles and semen quality.^17^ It is well known that oligospermia and weak sperm are the main causes of male infertility or fertility decline.^18^^,^^19^ The most common way to assess male fertility is to determine semen quality. Many studies have measured semen parameters in men with varicocele, and most patients have reduced semen quality.^20^, ^21^, ^22^ The present results show that except for semen pH, semen volume, concentration, total sperm count, normal form frequency, and total motility were lower in patients with varicocele. In this study, serum InhB and INSL-3 levels were combined with basic semen parameters to evaluate the degree of varicocele.

The hypothalamus-hypophysis-gonadal axis is a branch that controls the secretion of human sex hormones and is directly or indirectly involved in spermatogenesis and quality.^23^ Adult testes secrete testosterone, INSL-3, and InhB into the blood circulation, and these hormone levels are dynamically regulated.^24^ According to Chong et al., the correlation between testosterone and INSL-3 is low in normal young men.^25^ The same argument may apply to patients with varicocele. This study showed that although the testosterone of patients with varicocele was lower than that of normal controls, there was only a very weak negative correlation between testosterone and INSL-3, and no correlation with InhB. Leydig and Sertoli cells are activated by FSH, which can bind to Sertoli cells to establish a microenvironment for spermatogenesis.^26^ Leydig and Sertoli cells present negative feedback to the hypophysis and/or hypothalamus through their products testosterone and InhB, respectively, thus tightly regulating the gonadal axis.^12^ In the present study, FSH had a weak negative correlation with INSL-3 and InhB. In addition, serum INSL-3 also had a strong positive correlation with total sperm count, frequency of normal sperm morphology, and a weak positive correlation with total sperm motility frequency, while serum InhB showed a weak positive correlation with semen volume, sperm concentration, and total sperm count. The results indicated that FSH, INSL-3, and InhB may have similar negative feedback regulation axis, which may affect spermatogenesis and sperm quality.

InhB can be used as a serum marker of spermatogenesis, but its sensitivity and ability to predict spermatogenesis in different age groups is controversial. InhB has an inverted U-shaped relationship with age and is sensitive to the assessment of semen quality in men.^5^ The sensitivity of InhB reference range is poor when applied to patients with monorchidism.^27^ In this study, FSH was found to be slightly more correlated with semen quality than InhB. The results showed that both FSH and InhB could better reflect semen quality in patients with varicocele.

INSL3 is produced and secreted by the stromal cells of the testis.^28^ In animal studies, the stimulating effect of INSL-3 on testosterone secretion in stromal cells is realized through the activation of Camp.^29^ However, the present study did not find a correlation between INSL-3 and testosterone. However, the authors found a strong positive correlation between INSL-3 and semen quality, and the different severity of the disease in patients with varicocele was associated with serum INSL-3 levels. It is speculated that INSL-3 and testosterone production are independent under pathological conditions, and INSL-3 and semen quality can well evaluate the disease degree of patients with varicocele. The authors performed unsupervised PCA heat map analysis and found that serum InhB, INSL-3, and semen parameters could effectively distinguish between Grade II and Grade III varicocele.

## Conclusion

Semen parameters and the combination of serum InhB and INSL-3 levels in patients with varicocele are closely related to the severity of the disease. In particular, serum INSL-3 can well distinguish between Grade I and Grade II patients, which helps to understand the potential pathophysiological mechanism of varicocele and is expected to become a potential biomarker for early clinical intervention.

The datasets used and analyzed during the current study are available from the corresponding author upon reasonable request.

## CRediT authorship contribution statement

**Lei Liu:** Conceptualization, Methodology, Data curation, Writing – review & editing. **WenJie Huang:** Methodology, Data curation, Writing – review & editing. **KeBing Luo:** Supervision, Resources. **YiZhou Zeng:** Supervision, Resources. **YunHao Shao:** Validation. **ZongMin Long:** Validation.

## Declaration of competing interest

The authors declare no conflicts of interest.
